# Neural representations of visual statistical learning based on temporal duration

**DOI:** 10.1162/IMAG.a.135

**Published:** 2025-09-03

**Authors:** Sachio Otsuka, Jun Saiki

**Affiliations:** Graduate School of Human and Environmental Studies, Kyoto University, Sakyo-ku, Kyoto, Japan

**Keywords:** statistical learning, time perception, medial frontal gyrus, orbitofrontal cortex, multi-voxel pattern analysis

## Abstract

Time perception is an essential aspect of daily life, and transitional probabilities can be learned based on temporal durations that are independent of individual objects. Previous studies on temporal and spatial visual statistical learning (VSL) have shown that the hippocampus and lateral occipital cortex are engaged in learning visual regularities. However, it remains unclear whether VSL on temporal duration unlinked to object identity is represented in brain regions involved in VSL and object recognition or in those involved in time perception without sensory cortex involvement. We examined this question by adapting a VSL paradigm to time perception using functional magnetic resonance imaging. Thirty-four students participated in the VSL experiment, comprising a familiarization scan and a subsequent familiarity-decision test. The region-of-interest (ROI)-based classification showed chance-level performance across all ROIs, but only the left medial frontal gyrus, which is involved in subsecond time perception, showed a moderate effect size with 95% confidence intervals not crossing the chance level of 50%. Moreover, searchlight analysis showed that the right orbitofrontal cortex successfully decoded brain responses related to the processing of structured timing sequences. Meanwhile, representational similarity analysis suggested that the neural signal patterns could not be divided between the structured timing and pseudo-random sequences in the lateral occipital cortex. Our findings serve as a pilot study suggesting that the medial frontal and orbitofrontal regions are involved in VSL based on temporal duration, independent of visual object processing, which is a key and common timing mechanism for predicting sequential events.

## 1 Introduction

Time perception is essential in daily life. Learning the regularity of temporal information is necessary for language and motor acquisition, playing musical instruments, and traffic scenes (Grondin, 2010; Matthews & Meck, 2016; Merchant et al., 2013). For instance, in high traffic areas, one may need to predict the position of multiple targets within a time of a few hundred milliseconds to several seconds, in order to avoid collisions with people and bicycles. Some studies have reported that people are able to learn transitional probabilities based on the temporal duration of objects (Olson & Chun, 2001; Otsuka, 2023). Learning such regularities from the environment is called statistical learning (Fiser & Aslin, 2001, 2002; Frost et al., 2019; Saffran et al., 1996).

Saffran et al. (1996) were the first to show that 8-month-old infants could detect and learn statistical patterns from sequences of non-sensical syllables. Building on this foundational work, Fiser and Aslin (2001, 2002) conducted a series of studies with adult participants to examine statistical learning in spatial configurations and temporal sequences using visual stimuli. In a standard visual statistical learning (VSL) task focused on temporal structure, participants viewed a continuous stream of visual items during the familiarization phase. Certain triplets of items consistently appeared in the same order (e.g., ABC, DEF, GHI, and JKL). In the test phase, the participants completed a two-alternative forced choice (2AFC) familiarity task. Each test trial presented two sequences: a familiar triplet from the familiarization phase (e.g., ABC), and a foil composed of items drawn from three different triplets (e.g., AEI). The transitional probabilities within these foils were zero, based on the familiarization structure. Participants were asked to choose a sequence that seemed more familiar, and their performance indicated that they could reliably distinguish triplets from foils at levels significantly above chance (50%).

Previous studies of VSL have established that statistical learning can operate on various visual features, including letters, shapes, and colors (Bertels et al., 2015a, 2015b; Fiser & Aslin, 2002; Jeste et al., 2014; Turk-Browne & Scholl, 2009; Turk-Browne et al., 2005, 2008, 2009). This has also been observed for objects, faces, and natural scenes (Arciuli & Simpson, 2011a, 2011b; Brady & Oliva, 2008; Kim et al., 2014; Otsuka et al., 2016, 2024; Sherman & Turk-Browne, 2020; Turk-Browne et al., 2010). The strength of VSL depends on the duration of exposure to visual stimuli (Bertels et al., 2015b; Bogaerts et al., 2016; Turk-Browne et al., 2005). Furthermore, research has shown that learning temporal regularities can occur even when the object’s duration is unrelated to the object’s identity (Olson & Chun, 2001; Otsuka, 2023).

This study aimed to explore neural representations of VSL based on temporal duration. Specifically, we were challenged to clarify the question about the brain regions involved in VSL on temporal duration that is not linked to object identity by adapting the VSL paradigm to time perception. To achieve this, functional magnetic resonance imaging (fMRI) responses can be used to record while observing sequences with regularity in temporal duration and those with pseudo-random duration. Several fMRI studies have elucidated the neural basis underlying VSL. Turk-Browne et al. (2009) identified an enhanced response in the hippocampus of participants observing triplet sequences with temporal regularities, compared with the response to pseudo-random sequences (Karuza et al., 2017; Schapiro et al., 2014; Turk-Browne et al., 2010). Subsequently, the hippocampal regions were divided into the cornu ammonis (CA)1, CA3, dentate gyrus (DG), and subiculum (Schlichting et al., 2017; Sherman & Turk-Browne, 2020; Sherman et al., 2024), and it was reported that they are involved in VSL (Sherman & Turk-Browne, 2020). Moreover, the lateral occipital cortex (LOC), which is involved in object recognition, is activated during temporal and spatial VSL (Karuza et al., 2017; Turk-Browne et al., 2009). Notably, these brain regions are activated in situations where temporal or spatial regularities are linked to the event’s identity. In contrast, object duration was structured but object stimuli were presented in a random order in the VSL experiment conducted by Otsuka (2023). In other words, the observed VSL was based on temporal duration that was not linked to object identity. Therefore, it remains unclear whether VSL on temporal duration, which is not linked to object identity, is represented in brain regions involved in VSL and object recognition (Karuza et al., 2017; Sherman & Turk-Browne, 2020; Turk-Browne et al., 2009), or time perception with no sensory cortex involvement (Lewis & Miall, 2003; van Wassenhove et al., 2011; Wittmann et al., 2010). To the best of our knowledge, no studies have tested the neural mechanisms of VSL in terms of regularities based on time information.

Several studies have examined the neural bases of time perception. Wittmann et al. (2010) and van Wassenhove et al. (2011) showed that time was perceived longer for looming discs compared with receding discs and was significantly associated with activation in the medial frontal regions. Shih et al. (2009) reported that the supplementary motor area (SMA), which corresponds to Brodmann area (BA) 6, was commonly activated when estimating the duration of auditory stimuli and visual discs. In a recent meta-analytic study based on previous neuroscience studies of time perception, Nani et al. (2019) focused on whether time estimation involved a motor response and whether the estimation took less than or greater than 1 s. The resulting categories were as follows: subsecond (time estimation below 1 s) and motor; subsecond and nonmotor; suprasecond (time estimation above 1 s) and motor; and suprasecond and nonmotor. The results showed that the discrimination of different time intervals was supported by the SMA, including the medial frontal gyrus (MFG), middle frontal gyrus, and precentral gyrus, with the activation of these areas overlapping across the four abovementioned conditions. Thus, the SMA supporting time perception is predicted to be involved in VSL based on time information. A notable aspect of the existing literature pertains to the examination of time perception in response to alterations in the physical characteristics of objects, such as circles and lines, which are presented independently of their identity (Shih et al., 2009; van Wassenhove et al., 2011; Wittmann et al., 2010). Consequently, activation of the early visual cortex and LOC, which are involved in object recognition, was not observed.

In this study, multi-voxel pattern analysis (MVPA) was conducted with the aim of examining the neural representations of regularities on temporal duration. Based on previous neuroscience research on VSL and time perception, in the present study, the following brain regions were selected as regions-of-interest (ROIs): the subfield of the hippocampus involved in VSL (Sherman & Turk-Browne, 2020), regions involved in time perception without a motor response for both subsecond and suprasecond durations (Nani et al., 2019), and the LOC involved in object recognition (Malach et al., 1995; Turk-Browne et al., 2009). Given that these brain regions are involved in neural representations of VSL based on temporal duration, we hypothesized that MVPA performance would be superior to that obtained by chance. On the other hand, under the assumption that time-based VSL is represented independently of the visual information processing of objects, the hypothesis is that MVPA performance will be above chance level only in the ROIs related to VSL and time perception, whereas the LOC related to object recognition will show chance-level performance. Finally, we conducted a representational similarity analysis (RSA; Kriegeskorte et al., 2008; Kriegeskorte & Kievit, 2013) on the neural signal patterns to examine their similarity across ROIs.

## 2 Materials and Methods

### 2.1 Participants

An a priori power analysis of one-sample *t*-test was conducted (effect size *d* = 0.5, *α* = 0.05, and power = 0.80) using G* Power 3 (Version 3.1.9.6; Faul et al., 2007) and the R package *pwr* (Champely, 2020). This analysis suggested a required sample size of 34. Initially, 34 Japanese graduate and undergraduate students (24 men and 10 women aged between 20 and 29 years, mean age = 21.88 years, SD = 1.95) from Kyoto University participated in this experiment in exchange for a book coupon worth 5,000 Japanese yen. However, data from two participants were excluded because the operator at the MRI scanner and related facilities at the Institute for the Future of Human Society, Kyoto University noted that they had imaging evidence of brain atrophy, which was also confirmed by the first author. Therefore, two additional students were recruited to attain a sample size of 34. Finally, the data of 34 participants were used in the analysis (23 men and 11 women aged between 20 and 29 years, mean age = 21.91 years, SD = 1.96). All participants had normal or corrected-to-normal visual acuity with non-magnetic glasses and normal color vision based on the Ishihara color blindness test. In addition, all were right-hand dominant according to the H.N. Handedness Inventory (Hatta, 1975).

All participants provided written informed consent. All experimental protocols were approved by the Institutional Review Board of Kyoto University. This experiment was conducted at Kyoto University between October 29, 2021 and February 24, 2022.

### 2.2 Experimental stimuli and apparatus

Forty-eight objects were selected with properties described by Endo et al. (2003; Supplementary Fig. S1). Each object was presented eight times in the familiarization scan, and twice as triplets and foils in the subsequent test. The localizer scan involved 12 line drawings (apple, car, carrot, chair, dog, eagle, fly, gun, leg, piano, spoon, and window; Snodgrass & Vanderwart, 1980), 12 natural scenes (6 indoor scenes and 6 outdoor scenes; Xiao et al., 2010), and 12 faces (6 male faces and 6 female faces; Matsumoto & Ekman, 1988).

The objects and line drawings were presented in white, and natural scenes and faces in color, against a black background. To reduce eye strain in participants viewing visual stimuli inside an MRI scanner, we set the background color to black and the object color to white. Because an MRI scanner is a dark environment with blocked external light, setting the background white increases the overall brightness of the screen, which may cause visual discomfort or glare. By contrast, a black background suppressed the overall light intensity of the screen, ensuring sufficient contrast with the visual stimuli while minimizing the burden on the participants’ eyes. Therefore, to achieve both visibility and comfort in dark environments, we adopted a stimulus configuration in which white stimuli were presented on a black background. It has been shown that in low-light environments, dark mode (white text on a black background) reduces visual fatigue by promoting increased blink rate and improved pupil accommodation, which are indicators of visual fatigue (Xie et al., 2021). Visual stimuli were displayed on a screen of 1,024 × 768 resolution at the back of the MRI scanner bore, at a size that fell within the 256 × 256 range. Participants observed visual stimuli on a mirror attached to the head coil during fMRI. The experiment was run on a Windows laptop PC (ThinkPad X1 Extreme; Lenovo, Hong Kong, China) controlled by MATLAB (Version R2021a; The MathWorks, Inc., Natick, MA) and the Psychophysics Toolbox (Version 3.0.18; Brainard, 1997; Pelli, 1997). Behavioral responses within the MRI scanner were recorded using an MRI-compatible fiber-optic button box.

### 2.3 Procedure

To prevent the spread of coronavirus disease 2019 infection, participants cleaned their hands with alcohol disinfectant and had their body temperatures measured to confirm normothermia. Participants not wearing glasses wore a mask inside the MRI scanner, whereas those with non-magnetic glasses did not wear a mask inside the scanner to prevent their glasses from fogging up. All participants wore plastic gloves and a mask when performing the test task outside the MRI scanner.

The experiment consisted of familiarization scan, localizer scan, and the 2AFC familiarity-decision test outside the MRI scanner. In the familiarization scan, participants observed a visual stream containing 384 objects, using 48 object images. Object stimuli were presented in succession and in a random order at the center of the visual display with a white fixation point, to force the participants to fixate on the center of the screen. Specifically, the order of the 48 object images was randomized eight times, and they were combined with the restriction of not displaying the same object in succession. In this experiment, two sets of four structured triplets were made based on object duration. Specifically, 12 object durations were used in each set: 500, 600, 700, and 800 ms in the first group; 900, 1,000, 1,150, and 1,250 ms in the second group; and 1,350, 1,450, 1,550, and 1,650 ms in the third group of the first set; and 550, 650, 750, and 850 ms in the first group; 950, 1,050, 1,100, and 1,200 ms in the second group; and 1,300, 1,400, 1,500, and 1,600 ms in the third group of the second set. Half the participants observed the first set of durations in the structured block, and the second set in the pseudo-random block. The other half of the participants observed them vice versa. These durations were determined based on VSL research using time information (Otsuka, 2023). Specifically, the difference across durations within each set was set to 100 ms or more to make it as easy as possible for the participants to distinguish between the differences in object durations. Furthermore, the 12 durations in each set were divided into three groups such that the difference across the three durations within each triplet was as large as possible.

**Fig. 1.**
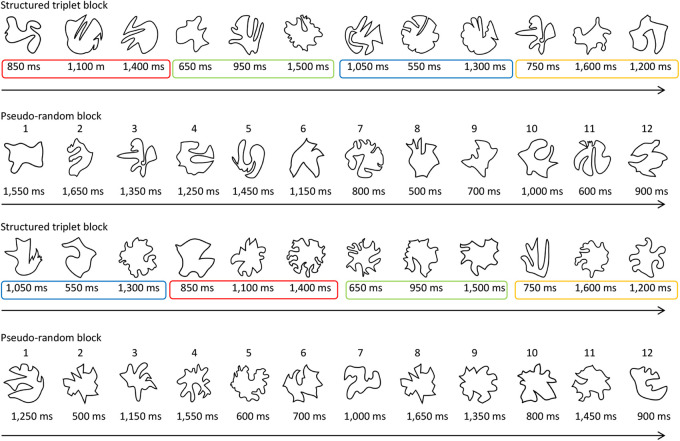
Examples from the structured and pseudo-random blocks provided to participants during a familiarization scan. These stimuli were selected from the stimulus set described by Endo et al. (2003). The colored rectangles represent structured triplets based on temporal duration, and the order of the three durations is always the same regardless of the object identity. Each timing triplet was presented once per structured block. The numbers shown on the objects represent their positions within the pseudo-random block. The ISI was dependent on the preceding object duration. For example, if an object duration was 900 ms, the subsequent blank duration would be 1,100 ms because the TR was 2,000 ms. In other words, the set of object stimuli and their blanks were presented at 2-s intervals in both blocks, in accordance with the scanner’s TR. Structured and pseudo-random blocks were presented in an alternating fashion, and the block order was counterbalanced across participants.

The familiarization scan had 32 × 30-s blocks (16 structured and 16 pseudo-random blocks), which were divided into four runs. A timing triplet was created by selecting one duration from each group and rearranging them in a random order (e.g., 850–1,100–1,400 ms, 1,050–550–1,300 ms, 750–1,600–1,200 ms, and 650–950–1,500 ms). For each participant, 12 durations were randomly assigned to each position of the four structured triplets (Supplementary Table S1). Thus, triplets were newly created for each participant and sets of three durations consistently appeared in identical order within each of the four structured triplets. However, objects were not linked to them in a visual sequence. Figure 1 illustrates the structured and pseudo-random triplets. For example, in a structured triplet block as indicated by the first line in Figure 1, the solid red, green, blue, and yellow lines represented the triplets consisting of three object durations. After an object was presented for 850 ms in a temporal triplet indicated by the solid red line, the next object was presented for 1,100 ms, and the next for 1,400 ms. The triplets shown in green, blue, and yellow were similar. Throughout the familiarization scan, this structure was preserved in the structured triplet block, except that the order of each triplet was random across the structured triplet blocks. Each timing triplet was presented once per structured block. The temporal durations of the three objects in the solid red, green, blue, and yellow lines were the same, as indicated by the third line in Figure 1.

To hold the same positional structure as in the structured triplets, the remaining set of 12 durations were assigned to one of four positions in the pseudo-random blocks (Turk-Browne, et al., 2009). More specifically, each duration was presented only in the corresponding position in the pseudo-random blocks. For example, when the durations in the first set of the pseudo-random blocks (1st, 4th, 7th, and 10th positions in each pseudo-random block) were 800, 1,000, 1,250, and 1,550 ms; those in the second set (2nd, 5th, 8th, and 11th positions) were 500, 600, 1,450, and 1,650 ms; and those in the third set (3rd, 6th, 9th, and 12th positions) were 700, 900, 1,150, and 1,350 ms (Supplementary Table S2), the pseudo-random sequences were created by randomly choosing one duration from each set in every pseudo-random block as follows: 1,550–1,650–1,350–1,250–1,450–1,150–800–500–700–1,000–600–900 ms in the first random block, as indicated by the second line in Figure 1; 1,250–500–1,150–1,550–600–700–1,000–1,650–1,350–800–1,450–900 ms in the second random block, as indicated by the fourth line in Figure 1; and so forth.

The interstimulus interval (ISI) was dependent on the preceding object duration. If an object duration was 900 ms, the subsequent blank duration would be 1,100 ms because the repetition time (TR) was 2,000 ms; this is explained further in the following ***fMRI data acquisition*** subsection. In other words, the set of object stimuli and their blanks were presented at 2-s intervals in both conditions, according to the scanner’s TR. Therefore, it is likely that such regularity was learned in both the structured and pseudo-random conditions. Subsequently, a 6,000-ms rest-period was inserted. In the familiarization scan, participants were asked to detect jiggling objects moving to the left and right by pressing the button with their right index finger as quickly and accurately as possible. Objects jiggled randomly once in each block. Structured and pseudo-random blocks were presented in an alternating fashion, and block order was counterbalanced across participants (Fig. 1). Participants were not informed that there was any structure in the visual stream of objects. A 10-s fixation block was also included before and after each stimulus presentation in each run, and the images of these fixation blocks did not contribute to the data analysis.

After the familiarization scan, to define the LOC related to object recognition (Malach et al., 1995; Turk-Browne et al., 2009), participants took part in one-run localizer scan. The localizer scan comprised 18 blocks, each involving the random presentation of 12 faces (6 male faces and 6 female faces), 12 natural scenes (6 indoor scenes and 6 outdoor scenes), or 12 line drawings (6 animal drawings and 6 vehicle drawings). In each block, a fixation cross was displayed for 6,000 ms with an instruction about the key configuration of the localizer task. Each stimulus was presented for 800 ms with a 1,200-ms ISI, resulting in 30-s blocks. Six blocks were included in each category, and the three categories (faces, natural scenes, and line drawings) alternated randomly every 30 s. Participants were asked to judge whether faces were male or female, whether scenes were indoors or outdoors, and whether drawings were animals or vehicles by pressing one of two buttons. In each block, the 6,000-ms fixation trial was inserted before the next block began.

Finally, participants were asked to take part in a 2AFC familiarity-decision test outside the MRI scanner, to examine whether they had learned the regularities of temporal duration during the familiarization scan. In each test trial, two three-object test sequences were shown at the center of the screen on a laptop PC. As in the familiarization scan, objects were presented successively in a random order. Specifically, 48 objects were randomized twice each for triplets and foils with the restriction that the same object was not presented in succession. One test sequence comprised a timing triplet containing temporal regularities of object duration in the familiarization scan (e.g., 850–1,100–1,400 ms, 1,050–550–1,300 ms, 750–1,600–1,200 ms, and 650–950–1,500 ms). The other was a foil constructed from object duration of three different triplets (e.g., 850–550–1,200 ms, 1,050–1,600–1,500 ms, 750–950–1,400 ms, and 650–1,100–1,300 ms). Thus, the transitional probabilities of object duration within foils were zero, based on the familiarization scan. After both test sequences were presented, participants were asked to indicate whether the first or the second test sequence seemed more familiar in the form of a forced choice between two options, based on the familiarization scan, by pressing either the “1” or “2” key on the laptop PC keyboard, respectively. Each of the four structured triplets was tested eight times and was paired twice with each of the four foils, resulting in a total of 32 test trials. In one trial, the first sequence was a structured triplet, and the second sequence was a foil (e.g., 850–1,100–1,400 ms vs. 850–550–1,200 ms). In the other trial, they were presented contrariwise (using the above example, 850–550–1,200 ms vs. 850–1,100–1,400 ms). The duration of each blank was 2 s minus the previous object duration. The two sequences were separated by a 1-s fixation point (Supplementary Fig. S2). In line with most previous studies (Brady & Oliva, 2008; Fiser & Aslin, 2002; Otsuka, 2023), participants’ performance in discriminating structured triplets from foils was used as a measure of VSL. After the 2AFC familiarity-decision test, participants were asked whether they noticed any structure in the visual stream during the familiarization scan.

### 2.4 fMRI data acquisition

The fMRI experiment was conducted using a 3-T Siemens scanner (3.0-T MAGNETOM Verio, Siemens Healthineers, Erlangen, Germany) at the Institute for the Future of Human Society, Kyoto University. Functional data were collected using a T2*-weighted gradient-echo, echo-planar imaging (EPI) sequence (echo time [TE] = 25 ms; TR = 2,000 ms; flip angle = 75°; matrix = 64 × 64; field-of-view = 224 mm; 3.5-mm thickness) with 34 axial slices. One-hundred and thirty volumes were acquired for each familiarization run, and 334 volumes for the localizer run: five volumes were discarded both before and after stimulus presentation. Structural images were acquired using a T1-weighted anatomical sequence (3-D magnetization-prepared rapid acquisition with gradient echo; TE = 3.51 ms; TR = 2,250 ms; flip angle = 9°; matrix = 256 × 256; 1.0 mm × 1.0 mm × 1.0 mm voxel size) after the second run of the familiarization scan.

### 2.5 Preprocessing

Preprocessing and statistical analyses were conducted with the Statistical Parametric Mapping (SPM) software (SPM 12 revision 7771, http://www.fil.ion.ucl.ac.uk) in MATLAB. Functional images were motion-corrected with realignment to the first image (interpolation comprised 2nd degree B-Spline in estimation and 4th degree B-Spline in reslice, with no wrapping), corrected for slice-acquisition time in ascending slice order after motion correction, and were coregistered using a rigid-body model. The functional images were then spatially normalized using the Montreal Neurological Institute (MNI) EPI template: the bias full-width at half-maximum was a 60-mm cutoff when estimated, and interpolation was 4th degree B-Spline when written. However, brain images were not smoothed for the MVPA. Structural images were segmented with three tissue classes: grey matter, white matter, and cerebro-spinal fluid.

### 2.6 Data analyses

#### 2.6.1 Familiarization scan

To examine for differences in neural responses with respect to the structured and pseudo-random sequences, a general linear model (GLM) analysis was conducted using SPM. Two event types for the sequences (the structured and pseudo-random sequences) were entered as separate regressors. The rest period between the structured and pseudo-random blocks was also entered as a regressor. The durations of each sequence were set as 24 s, and those of a rest period as 6 s. Regardless of whether the participants detected jiggling objects during the familiarization scan, all images in all blocks were used for the following analysis. Moreover, six regressors for each dimension of head motion were included as covariates of no interest. Each run was high-pass filtered with a 128-s cutoff to remove low-frequency noise. Canonical hemodynamic response function was used with no derivatives. This model estimated the contribution of each condition to the BOLD response in every voxel for each participant (beta values). The fMRI responses were used in the subsequent classification analysis.

The Decoding Toolbox (Version 3.999E or later; Hebart et al., 2015) and a linear support vector machine with a cost parameter of 1 as a classifier were used in the MVPA. MVPA is a method used to investigate the information represented in the brain by analyzing the spatial patterns of signals across multiple voxels. Even when stimuli A and B activate the same brain region to a similar degree, the spatial patterns of the voxel signals in that region may differ. Through the application of machine learning techniques, MVPA is able to identify differences in spatial patterns exhibited by multiple voxels (Mur et al., 2009).

In some VSL studies, the hippocampal regions were divided into the CA1, CA3, DG, and subiculum (Schlichting et al., 2017; Sherman et al., 2024), which have been reported to be involved in VSL (Sherman & Turk-Browne, 2020). The Advanced Normalization Tools in Python – Neural Network Toolkit (ANTsPyNet; Tustison et al., 2021) was used to create ROI images by dividing each participant’s bilateral hippocampus into the head of the CA1, CA3, DG, and subiculum. ANTsPyNet is a deep learning-based brain image analysis toolkit built on top of ANTsPy, which is a Python-based medical image processing library. It specializes in preprocessing brain MRI images and segmenting brain regions. Specifically, the *antspynet.deep_flash function* was used to create ROI images for the bilateral head of the DG/CA3, CA1, and subiculum using label numbers 13, 14, 15, 16, 17, and 18, respectively.

Voxel-wise fMRI responses in the ROIs were extracted during the familiarization scan in the structured and pseudo-random conditions. Each ROI was defined in line with previous studies of VSL and time perception as follows: the bilateral head of CA1, CA3/DG, and subiculum of hippocampus related to VSL (Sherman & Turk-Browne, 2020); the left MFG (–4, –21, 55), the right SFG (2, 8, 55), left precentral gyrus (–46, –2, 35), and left middle frontal gyrus (–24, –11, 54) related to subsecond time perception (Nani et al., 2019); the left MFG (–6, 1, 57), right SFG (6, 8, 59), right precentral gyrus (38, 0, 35), and right middle frontal gyrus (30, 14, 55) related to suprasecond time perception (Nani et al., 2019). The ROI images related to subsecond and suprasecond time perception were created with an 8-mm-radius sphere ROI, with reference to Barbeau et al. (2023). The ROIs related to time perception were selected using the following criteria: 1) areas present in both subsecond and suprasecond time perception without motor responses, and 2) in BA 6 that incorporates the SMA. Classification accuracies were calculated in each ROI using the Decoding Toolbox (Hebart et al., 2015) with leave-one-run-out cross-validation. A classifier was trained to discriminate between structured and pseudo-random sequences within the brain activity data in three runs. Subsequently, this classifier was tested on its ability to successfully discriminate the data of the remaining run that had not been included in the training. If each ROI encoded significantly discriminable neural representations of the above two sequences, the corresponding classifier would be expected to learn the patterns of brain activities during the training and to predict the sequences accurately in the test data. The above procedures were repeated for all four familiarization scan runs, and the data were used to determine mean classification accuracies across runs for each participant. A one-sample *t* test (two-tailed) or Wilcoxon signed-rank test was used to examine whether the mean accuracies across participants were above the chance level (50%) for each ROI using the Benjamini-Hochberg procedure. If neural activity within each ROI represented discriminable patterns between the structured and pseudo-random conditions, classification accuracies would be expected to exceed chance level.

To examine how similar the patterns of estimated fMRI signals are between the structured timing and pseudo-random blocks across the voxel sets in each ROI, an RSA was conducted (Kriegeskorte et al., 2008; Kriegeskorte & Kievit, 2013). First, we calculated the z-scored correlation matrices of the estimated fMRI signals in each ROI using the Decoding Toolbox (Hebart et al., 2015). Then, the 8 × 8 correlation matrix values with two conditions (structured and pseudo-random conditions) and four runs were vectorized. Distance matrices (Euclidean distance) were calculated from the correlation matrices to examine the similarity of the neural representations of the structured and random conditions. Specifically, the data of the distance matrices were compressed into two dimensions and visualized by t-distributed Stochastic Neighbor Embedding (t-SNE; van der Maaten & Hinton, 2008).

To explore other regions that may represent VSL based on temporal duration, a searchlight analysis was performed on the imaging data of each participant, with a 4-mm-radius. The searchlight analysis images of each participant were normalized and spatially smoothed using an 8-mm Gaussian kernel. Finally, a second-level analysis using SPM was performed on each participant’s data, using behavioral performance in the familiarity-decision task as a covariate. Voxels were judged to show a reliable difference if the *t* values reached a cluster-level significance threshold of *p* < 0.05, family-wise error corrected. This was achieved by first applying a voxel-level threshold of *p* < 0.001 (uncorrected), followed by identifying contiguous clusters of voxels exceeding this threshold. The statistical significance of these clusters was then determined using a cluster-size threshold calculated based on a family-wise error rate of *p* < 0.05, which accounts for multiple comparisons across the entire brain volume (see Lieberman & Cunningham, 2009; Woo et al., 2014).

#### 2.6.2 Localizer scan

To examine whether VSL based on temporal duration was represented in the brain regions related to object recognition, the LOC was localized on imaging across participants. Specifically, the same preprocessing performed on the data of the familiarization scan was conducted on the data of the localizer scan, except that images obtained for the latter were spatially smoothed using an 8-mm Gaussian kernel. Then, an additional GLM analysis was conducted with the following regressors: line drawings, natural scenes, faces, instruction, and rest period. The durations of each category were set as 24 s, whereas the durations of an instruction or rest period were 6 s. Six regressors for each dimension of head motion were also included. Functional images in the blocks of line drawings were contrasted with those of faces. The statistical analysis was conducted using SPM’s first-level analysis framework. To determine whether the observed differences were statistically reliable compared to chance level, a cluster-level significance threshold of *p* < 0.05, family-wise error corrected, was applied, as used in the searchlight analysis. As the LOC could not be localized in some participants (15 participants), the coordinates of the right and left LOC were used based on the results of the group-level analysis (right: 30, –91, 5; left: –21, –91, –4; Supplementary Fig. S3). Average accuracies in the MVPA (i.e., the mean of the right and left LOC) were compared with chance level.

### 2.7 Statistical analysis

R (Version 4.4.2; R Core Team, 2024) and RStudio software (Version 2024.12.0 + 467; RStudio Team, 2024) was used to conduct both standard *t*-tests and non-parametric tests. Additionally, the R package *exactRankTests* (Version 0.8-35; Hothorn & Hornik, 2022) was used to conduct the Wilcoxon signed-rank test. Cohen’s *d* (by applying the Hedges correction) was reported using the R package *effectsize* (Version 1.0.0; Ben-Shachar et al., 2020). The results were visualized using the R packages *ggplot2* (Version 3.5.1; Wickham, 2016) and *ggthemes* (Version 5.1.0; Arnold et al., 2024). In addition, the following R packages were used in this study: *abind* (Version 1.4-8; Plate & Heiberger, 2024), *ggbeeswarm* (Version 0.7.2; Clarke et al., 2023), *magick* (Version 2.8.5; Ooms, 2024), *Matrix* (Version 1.7-2; Bates et al., 2025), *psych* (Version 2.4.12; Revelle, 2024), *pwr* (Version 1.3-0; Champely, 2020), *reshape2* (Version 1.4.4; Wickham, 2020), *Rtsne* (Version 0.17; Krijthe, 2023), *stylo* (Version 0.7.5; Eder et al., 2024), and *tidyverse* (Version 2.0.0; Wickham, 2023). Bayesian *t*-tests and non-parametric tests (prior = Cauchy, r = 0.707) were conducted by the JASP software (Version 0.19 or later; JASP Team, 2024).

Requests for the data used in behavioral and decoding results and analysis code can be sent via email to the first author. This study was not pre-registered.

## 3 Results

### 3.1 Behavioral results

**Fig. 2.**
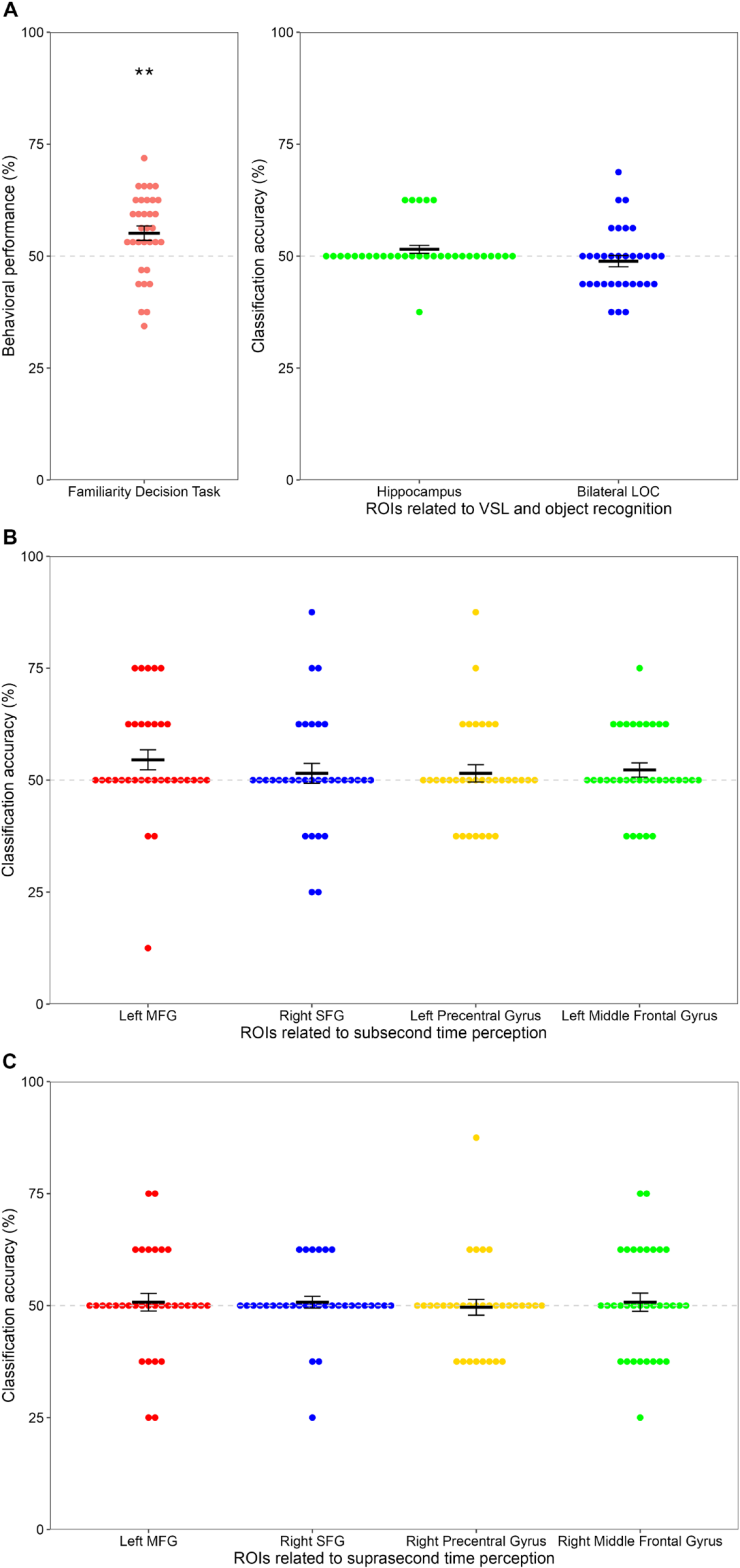
Behavioral results in the two-alternative forced-choice familiarity-decision task and averaged classification accuracies during the familiarization scan in the ROIs (*N* = 33). (A) Behavioral performance in the familiarity-decision task (left panel) and classification accuracies in the hippocampus and bilateral LOC, which are related to VSL and object recognition (right panel). (B) Classification accuracies in the left MFG, right SFG, left precentral gyrus, and left middle frontal gyrus, which are related to subsecond time perception. (C) Classification accuracies in the left MFG, right SFG, right precentral gyrus, and right middle frontal gyrus, which are related to suprasecond time perception. The grey dotted line indicates chance-level accuracy (50%), and error bars represent the standard error. Each point represents an individual score. LOC: lateral occipital cortex; ROIs: regions of interest; VSL: visual statistical learning; MFG: medial frontal gyrus; SFG: superior frontal gyrus. Asterisks indicate significant differences from chance-level performance (**p < 0.01).

One female participant (age = 21 years) showed behavioral performance less than 70% in the familiarization scan. According to the criteria reported in Otsuka et al. (2024), the data of this participant were excluded from both the behavioral and fMRI data analyses. The remaining 33 participants (23 men and 10 women, aged 20–29 years, mean age = 21.94 years, standard deviation [SD] = 1.98) were able to detect 70% or more of the jiggling objects (97.35%, standard error [SE] = 1.14; mean number of false alarms = 0.94, SE = 0.42), demonstrating that they correctly followed the instructions and observed the visual stream of objects. A one-sample *t*-test was conducted to compare the proportion of triplets judged as familiar in the 2AFC familiarity-decision test to chance level (50%). Structured triplets could be significantly discriminated from foils (55.11%, SE = 1.60), *t*(32) = 3.20, *p* = 0.003, *BF*_10_ = 12.04, *d* = 0.54, 95%CI [51.86, 58.37], suggesting that participants learned the regularities of structured triplets based on the temporal duration of objects observed during the familiarization scan (Fig. 2A, left panel). Following the experiment, none of the participants reported noticing temporal regularities during the familiarization scan.

### 3.2 ROI-based MVPA results

The data of the above-mentioned 33 participants were also used in the MVPA. The MVPA was conducted to explore whether the regions engaged in time perception (Nani et al., 2019), VSL (Sherman & Turk-Browne, 2020), and object recognition (Malach et al., 1995; Turk-Browne et al., 2009) encoded the representations of VSL based on temporal duration. The classification analysis between the structured and pseudo-random sequences during the familiarization scan revealed that chance performances were observed in all ROIs, using a Wilcoxon signed-rank test with the Benjamini-Hochberg correction as a Shapiro–Wilk test showed a non-normal distribution.

The classification accuracy for the hippocampus, which is linked to VSL, was 51.52% (SE = 0.90), *V* = 17.50, *p* = 0.802, *BF*_10_ = 0.39, *r* = 0.21, 95%CI [37.50, 62.50]. Similarly, the ROIs related to subsecond time perception showed no above-chance performance. The values were as follows: left MFG, 54.55% (SE = 2.23, *V* = 95.00, *p* = 0.408, *BF*_10_ = 1.39, *r* = 0.36, 95%CI [50.00, 75.00]); right superior frontal gyrus (SFG), 51.52% (SE = 2.23, *V* = 62.00, *p* = 0.826, *BF*_10_ = 0.23, *r* = 0.09, 95%CI [37.50, 68.75]); left precentral gyrus, 51.52% (SE = 1.94, *V* = 71.00, *p* = 0.826, *BF*_10_ = 0.23, *r* = 0.10, 95%CI [37.50, 62.50]); and left middle frontal gyrus, 52.27% (SE = 1.58, *V* = 82.50, *p* = 0.802, *BF*_10_ = 0.43, *r* = 0.20, 95%CI [37.50, 62.50]). For the ROIs related to suprasecond time perception, the results were again at chance level: left MFG, 50.76% (SE = 1.96, *V* = 58.00, *p* = 0.826, *BF*_10_ = 0.21, *r* = 0.04, 95%CI [37.50, 62.50]); right SFG, 50.76% (SE = 1.33, *V* = 27.00, *p* = 0.826, *BF*_10_ = 0.27, *r* = 0.05, 95%CI [37.50, 62.50]); right precentral gyrus, 49.62% (SE = 1.76, *V* = 39.00, *p* = 0.826, *BF*_10_ = 0.22, *r* = 0.04, 95%CI [37.50, 62.50]); and right middle frontal gyrus, 50.76% (SE = 1.76, *V* = 104.00, *p* = 0.826, *BF*_10_ = 0.22, *r* = 0.05, 95%CI [43.75, 62.50]). Finally, in the bilateral LOC, which is related to object recognition, the classification accuracy was 48.86% (SE = 1.26, *V* = 89.00, *p* = 0.826, *BF*_10_ = 0.30, *r* = 0.17, 95%CI [40.63, 53.13]) (Fig. 2A, right panel, Fig. 2B, C; Table 1).

**Table 1.** Summary of ROI-based MVPA.

*ROIs involved in VSL*									
*Region*	*Hemisphere*	*x*	*y*	*z*	*MVPA scores (SE)*	*p values*	*BF_10_*	*Effect size*	*95%CI*
Hippocampus	Bilateral				51.52 (0.90)	0.802	0.39	0.21	[37.50, 62.50]

*ROIs involved in subsecond time perception*									
*Region*	*Hemisphere*	*x*	*y*	*z*	*MVPA scores (SE)*	*p values*	*BF_10_*	*Effect size*	*95%CI*
MFG	Left	–4	–21	55	54.55 (2.23)	0.408	1.39	0.36	[50.00, 75.00]
SFG	Right	2	8	55	51.52 (2.23)	0.826	0.23	0.09	[37.50, 68.75]
Precentral gyrus	Left	–46	–2	35	51.52 (1.94)	0.826	0.23	0.10	[37.50, 62.50]
Middle frontal gyrus	Left	–24	–11	54	52.27 (1.58)	0.802	0.43	0.20	[37.50, 62.50]

*ROIs involved in suprasecond time perception*									
*Region*	*Hemisphere*	*x*	*y*	*z*	*MVPA scores (SE)*	*p values*	*BF_10_*	*Effect size*	*95%CI*
MFG	Left	–6	1	57	50.76 (1.96)	0.826	0.21	0.04	[37.50, 62.50]
SFG	Right	6	8	59	50.76 (1.33)	0.826	0.27	0.05	[37.50, 62.50]
precentral gyrus	Right	38	0	35	49.62 (1.76)	0.826	0.22	0.04	[37.50, 62.50]
Middle frontal gyrus	Left	30	14	55	50.76 (2.03)	0.826	0.22	0.05	[43.75, 62.50]

*ROIs involved in object recognition*									
*Region*	*Hemisphere*	*x*	*y*	*z*	*MVPA scores (SE)*	*p values*	*BF_10_*	*Effect size*	*95%CI*
LOC	Right	30	–91	5					
	Left	–21	–91	–4	48.86 (1.26)	0.826	0.30	0.17	[40.63, 53.13]

*x*/*y*/*z*, ROI coordinates in MNI space; ROIs: regions of interest; VSL: visual statistical learning; MVPA: multi-voxel pattern analysis; BF: Bayes Factor; CI: confidence interval; MFG: medial frontal gyrus; SFG: superior frontal gyrus; LOC: lateral occipital cortex.

The MVPA results showed chance performance in all predefined ROIs, but a moderate effect size was observed only in the left MFG, which is involved in subsecond time perception, and the 95% confidence interval did not cross the chance level of 50%.

### 3.3 RSA

**Fig. 3.**
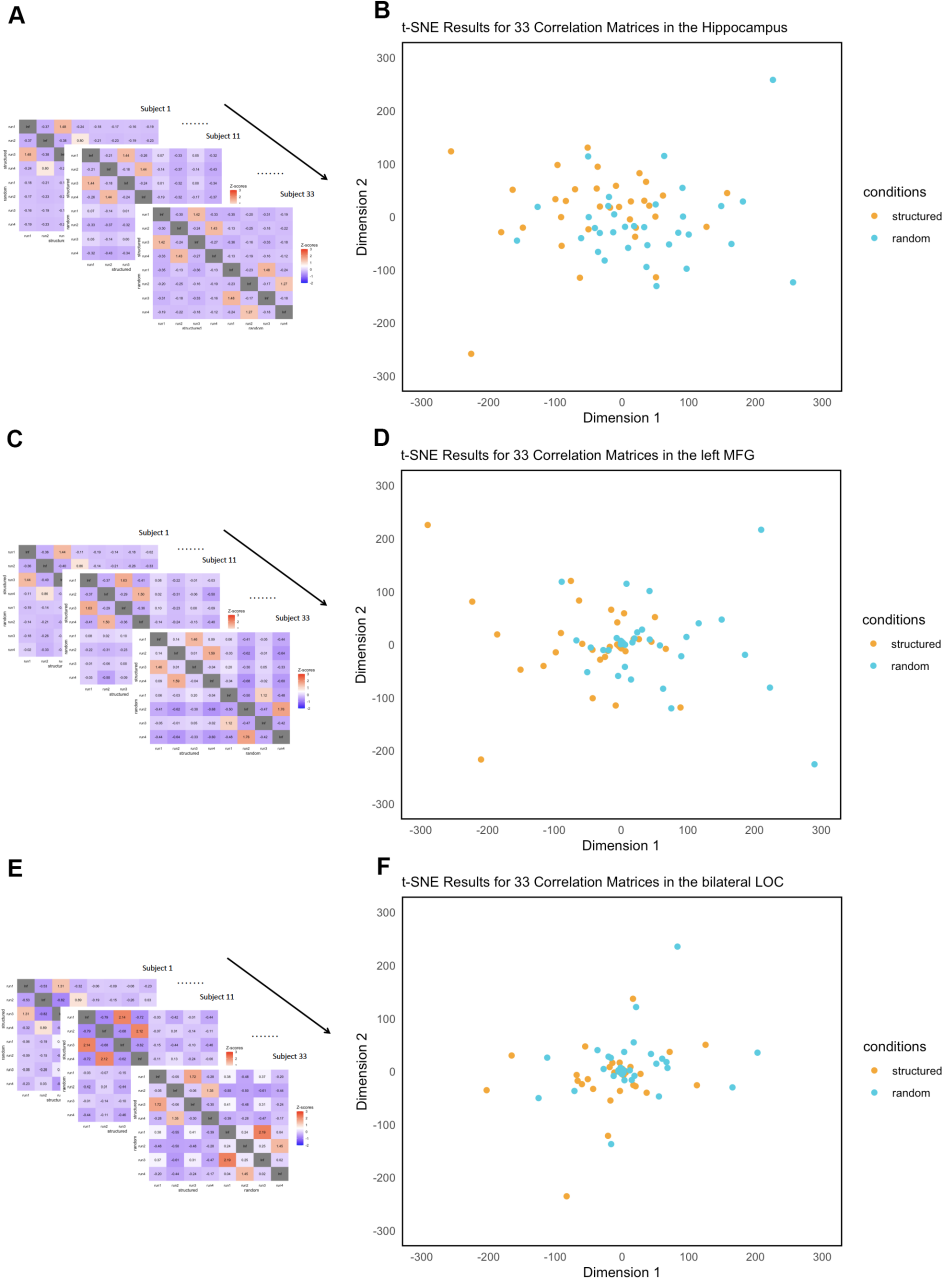
Results of the representational similarity analysis in the hippocampus related to VSL, the left MFG related to subsecond time perception, and the bilateral LOC related to object recognition. (A, C, E) Z-scored correlation matrices of fMRI data during the familiarization scan. (B, D, F) Visualization of fMRI data as a function of the structured and pseudo-random conditions during the familiarization scan using t-SNE. Each point represents an individual score. The RSA showed that the difference between structured and pseudo-random conditions could be found in the left MFG, but not in the right hippocampus and bilateral LOC. MFG: medial frontal gyrus; LOC: lateral occipital cortex; ROIs: regions of interest; t-SNE: t-distributed Stochastic Neighbor Embedding; VSL: visual statistical learning.

To further examine the similarities in neural patterns in fMRI signals between structured and pseudo-random conditions in the hippocampus, the left MFG related to subsecond time perception, and the LOC, an RSA was conducted (Kriegeskorte et al., 2008; Kriegeskorte & Kievit, 2013). MVPA primarily focuses on distinguishing or classifying specific conditions or stimuli based on neural activity patterns. In contrast, RSA analyzes the similarity between neural activity patterns to understand the structure of information represented in the brain. Specifically, z-scored correlation matrices of the estimated fMRI signals were calculated in each ROI (Fig. 3A, C, E). t-SNE (van der Maaten & Hinton, 2008) visually showed that data from the bilateral LOC were similar in the structured and pseudo-random conditions (Fig. 3F), whereas they were relatively far apart in the hippocampus and left MFG (Fig. 3B, D).

Furthermore, we examined whether the clusters under the structured and pseudo-random conditions could be separated based on the t-SNE results, and statistically tested whether the separation was due to chance using a permutation test (iteration = 1,000). Specifically, we calculated the average vector-to-vector distance in the t-SNE space between the structured and pseudo-random conditions, compared it with the average distance distribution obtained by shuffling these labels, and tested whether the observed distance was significantly greater than the shuffled distance. The results of the permutation test with the Benjamini-Hochberg correction were significant for the hippocampus and left MFG (*p*’s = 0.041), but not for the LOC (*p* = 0.289).

### 3.4 Searchlight MVPA results

**Fig. 4.**
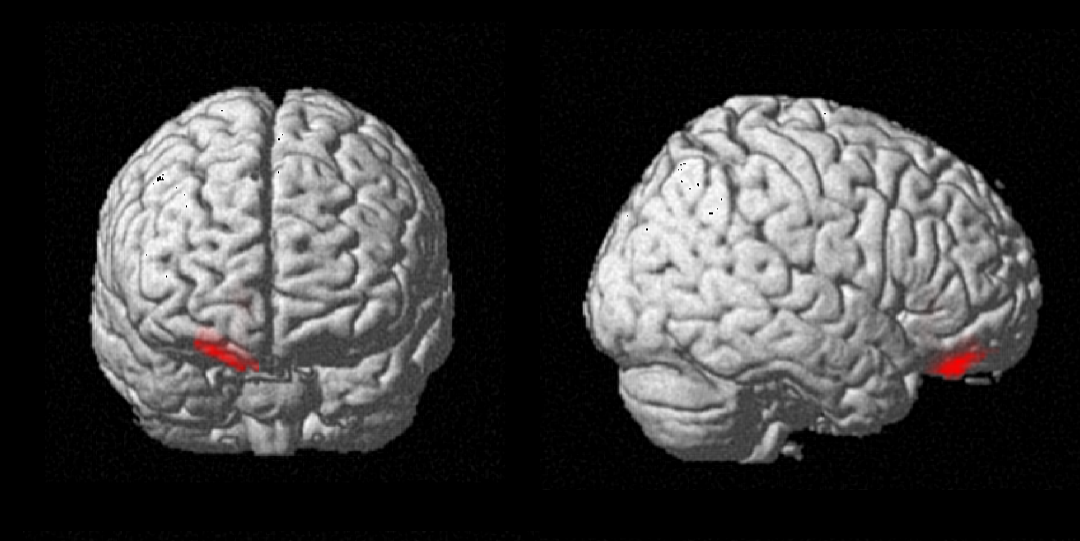
Results of a searchlight analysis of the structured versus pseudo-random sequences during the familiarization scan. The right orbitofrontal cortex exhibited an MVPA accuracy above chance level (24, 44, –18; *p* = 0.013 corrected; *k* = 563; peak *t* = 4.70). MVPA: multi-voxel pattern analysis. No masks were used, and only the areas that were significant at the cluster level threshold were displayed.

Finally, a searchlight analysis was conducted to explore other regions that may represent VSL based on temporal duration. The results showed significantly above-chance performance only in the clusters centered on the right orbitofrontal cortex (Fig. 4: OFC; 24, 44, –18; *p* = 0.013 corrected; extent = 563; peak *t* = 4.70); however, no significant correlations were observed between behavioral performance in the familiarity-decision test and the brain areas. The RSA was also conducted on the right OFC, which showed that structured and pseudo-random triplets were represented differently, in the same way as shown in the left MFG involved in subsecond time perception (Supplementary Figs. S4 and S5). Furthermore, using a permutation test, we compared the average vector distances in the t-SNE space under structured and pseudo-random conditions with the average distance distribution obtained by shuffling the labels. The results showed that the observed distances were significantly greater than the shuffled distances (*p* = 0.029).^*^

## 4 Discussion

The current study demonstrated that performance in the familiarity-decision task to structured timing triplets was significantly better than that expected by chance. The behavioral results suggest that people can extract and learn statistical regularities based on object temporal duration (Olson & Chun, 2001; Otsuka, 2023). Although the results were statistically significant, they were close to chance level. The behavioral performance reported in some fMRI studies using VSL indicates chance levels (Sherman & Turk-Browne, 2020; Turk-Browne et al., 2009). This is likely due to the fact that, similar to the present study, the stimuli presented during the familiarity phase were not limited to triplets with regularity but included random sequences without regularity.

### 4.1 ROI-based classification analysis and RSA: Neural representations of VSL based on object duration in the MFG

MVPA was performed on voxel responses while observing structured triplet blocks with regular object durations and pseudo-random blocks. However, the one-sample test with multiple comparisons showed a chance performance for all ROIs. In this study, 10 ROIs were predefined based on previous studies on VSL, time perception, and object recognition (Malach et al., 1995; Nani et al., 2019; Sherman & Turk-Browne, 2020; Turk-Browne et al., 2009). When multiple comparison corrections are applied, especially with strict methods such as the Bonferroni correction, the statistical power is greatly reduced in fMRI studies (Lieberman & Cunningham, 2009). If the effect size for each ROI is small, there is a possibility that the true effect will be missed by applying corrections. Therefore, we focused on effect sizes and 95% confidence intervals for MVPA performance in each ROI. Moderate effect sizes and confidence intervals that did not cross 50% performance were observed only in the left MFG, which is involved in subsecond time perception. In contrast, small effect sizes and confidence intervals that crossed 50% performance were observed in all other ROIs. Furthermore, we evaluated the separation between clusters under structured and pseudo-random conditions based on the t-SNE results. The results showed that the separation was statistically significant in the left MFG. These results suggest that the left SMA within the MFG represents temporal information of statistical regularities, which is in line with the results of the aforementioned meta-analysis (Nani et al., 2019) that evaluated previous data on time perception (Herbst et al., 2022; Lewis & Miall, 2003; Schubotz & von Cramon, 2001; Shih et al., 2009; van Wassenhove et al., 2011; Wittmann et al., 2010).

In contrast, LOC involvement in VSL based on object duration was not found in the ROI-based classification analysis, with a small effect size and a 95% confidence interval crossing the 50% chance level. The RSA and permutation test results also showed that the neural patterns of structured timing and pseudo-random sequences could not be separated in the bilateral LOC. These results are inconsistent with the findings of previous VSL studies (Karuza et al., 2017; Turk-Browne et al., 2009). These differences may be explained by the fact that in the present study, object identity was not linked to the regularity of object duration. Some neuroscience studies on visual time perception have not observed activations in the early visual cortex or LOC, and the results of this study are consistent with them (Lewis & Miall, 2003; van Wassenhove et al., 2011; Wittmann et al., 2010).

MVPA performance reached the chance level in the hippocampal subfield, with a small effect size and a 95% confidence interval crossing the 50% chance level; however, the results of RSA and permutation tests indicated that neural signals under structured and pseudo-random conditions may be represented separately in the hippocampal region. These results suggest that VSL based on temporal information is supported by the hippocampus. This is comparable with that of a study by Harrington et al. (2004), which indicated the involvement of memory in evaluating time intervals on the activations of the right hippocampal and left medial frontal areas during interval encoding. However, given the observed discrepancies between the MVPA and RSA results, the neural representations of VSL based on temporal durations in the hippocampal region remain uncertain. Further empirical research is required to address this issue.

The classification results showed a chance-level performance, a small effect size, and a 95% confidence interval crossing the 50% chance level in the left MFG ROI, where activation was observed in suprasecond time perception (Nani et al., 2019). Morillon et al. (2009) reported that the anterior SMA/SMA was activated in a time-evaluation task relative to a color discrimination task. One possible explanation for these contradictory results in this brain region is that the participants of our study did not attend to the temporal duration of objects. In this study, participants were not instructed to pay attention to object duration while observing the object sequence, as in a simple reaction-time task used in time-perception studies (Capizzi & Correa, 2018; Nobre & van Ede, 2018). This prevented the participants from consciously noticing regularities based on the object duration by paying attention to them. Given that the participants were not instructed to attend to the time information, MVPA performance might have reached a chance level for all predefined ROIs. In a recent study that incorporated high-definition transcranial random noise stimulation (Capizzi et al., 2023), SMA involvement was observed in a task that explicitly directed attention to the target duration but not in an implicit temporal-evaluation task. Moreover, Mondok and Wiener (2023) showed, by reverse inference using Bayes factor modelling, that explicit time perception was associated with the highest activations of posterior probability in the left SMA. Alternatively, although this study used a mixture of subsecond and suprasecond object durations, the differences in these durations within triplets were mostly subsecond. Therefore, VSL based on object duration might not have been represented in the left MFG that is associated with suprasecond time perception. Future studies should examine whether VSL based on temporal information, especially in suprasecond time perception, is represented in the SMA when participants receive explicit instructions on temporal regularities.

### 4.2 Searchlight analysis: Neural representations of VSL based on object duration in the OFC

An exploratory searchlight analysis revealed that the right OFC exhibited higher MVPA performance than chance level, thereby suggesting that the right OFC is involved in VSL based on object duration. The OFC is associated with reward prediction and expectation (Bechara et al., 1994; Gottfried et al., 2003; Kringelbach, 2005; Roesch et al., 2006; Tremblay & Schultz, 1999), as well as object anticipation in the perception of objects and natural scenes (Bar, 2003, 2004; Peelen et al., 2024). Furthermore, the OFC plays an important role in reward encoding and temporal discounting, with different functions of reward value and its relation to time identified in the medial and lateral OFC (Izquierdo, 2017). Based on the role of the OFC in reward encoding and time perception (Mazur & Biondi, 2009; Sellitto et al., 2010), Sosa et al. (2021) recently proposed that the OFC contributes to integration of reward magnitude and delay. In the current study, the structured triplet blocks allowed participants to anticipate the subsequent duration after learning the regularities of object duration, but no reward or value was provided to them for doing so. Therefore, the neural representations related to VSL based on object duration observed in the right OFC are independent of reward or value. However, no correlation was found between searchlight MVPA performance on the OFC and behavioral performance on the familiarity decision test. It should also be noted that the OFC was not a predefined ROI.

In this study, we did not directly measure eye movements; however, we presented the fixation point to the participants and instructed them to fixate on it. Therefore, we believe that the influence of eye movements was controlled to a certain extent. However, it is still possible that the influence of eye movements cannot be eliminated. Our findings raise the possibility that the OFC may be involved in VSL based on temporal duration, although alternative explanations such as potential signal dropout and unmeasured eye movements cannot be ruled out. The OFC is structurally susceptible to the influence of magnetic susceptibility artifacts and is prone to fMRI signal dropouts (Deichmann et al., 2003; Devlin et al., 2000; Ojemann et al., 1997). Consequently, a potential bias may have emerged in the number of valid voxels across the participants or conditions. Such a voxel number imbalance could have influenced the classifier’s learning, and it cannot be completely ruled out that the classification accuracy exceeded chance level by chance. When assessing the performance of classification systems in the OFC, it is imperative to exercise caution and consider the constraints imposed by signal quality.

### 4.3 Limitations of the current study

Although this study determined the ROIs involved in time perception based on a previous meta-analytic study (Nani et al., 2019), the basal ganglia (Shih et al., 2009), cerebellum (van Wassenhove et al., 2011), and other regions involved in time perception except for BA 6 might represent VSL based on object duration. Moreover, this study selected the LOC as the ROI for object recognition considering temporal and spatial VSL studies (Karuza et al., 2017; Turk-Browne et al., 2009). However, it remains unclear whether other brain regions involved in object recognition, such as the fusiform gyrus (Kanwisher et al., 1996) and the early visual cortex, represent VSL based on temporal duration. Turk-Browne et al. (2009) reported that the fusiform gyrus was activated during VSL and that the activation in the right hippocampus related to anticipation was negatively correlated with that in the early visual cortex involved in processing of predicted items (Turk-Browne et al., 2010). Therefore, it is necessary to examine whether VSL based on temporal information is also represented in other brain regions involved in object recognition in the future.

As described in the Materials and Methods section, an additional regularity was implemented in both the structured and pseudo-random conditions, whereby visual objects appeared at 2-s intervals based on the TR of the MRI device. Therefore, a distinction between structured and random conditions was founded on this additional regularity. Consequently, the participants were exposed to a certain type of temporal regularity throughout the experiment, wherein objects were presented at 2-s intervals in all blocks, potentially engendering a situation conducive to extracting and learning temporal regularity. Weak neural representations of temporal regularity observed in the left MFG may be contingent on the characteristics of the MRI device.

Considering the previous findings of lower behavioral performance for VSL based on object duration (Otsuka, 2023), compared with temporal VSL when using objects and natural scenes (Brady & Oliva, 2008; Fiser & Aslin, 2002; Otsuka et al., 2024), this study employed a block design instead of an event-related design to capture larger brain activations. Unlike an event-related design, which has been utilized in previous VSL studies (Sherman & Turk-Browne, 2020; Turk-Browne et al., 2010), a block design procedure is unable to capture brain activities for individual triplets, predictive events, and predicted events. Therefore, a block design might have obscured the neural representations associated with VSL based on object duration in the hippocampus and LOC. Future studies should evaluate whether VSL based on object duration is represented in the hippocampus and LOC using an event-related design as well as whether similar results are obtained when replicating the experiments.

## 5 Conclusion

The results of the ROI-based MVPA, including classification analysis and RSA, suggest that VSL based on object duration is represented in the MFG, which is reportedly involved in subsecond time perception, without LOC involvement. Moreover, a searchlight analysis indicated that it is also represented in the OFC, which has been implicated in reward prediction and object anticipation. Our findings serve as a pilot study suggesting that the SMA and OFC are involved in the key neural mechanisms underlying learning regularities based on temporal duration, which is consistent with a common timing mechanism related to time perception (Grondin, 2010; Merchant et al., 2013). Neural representations of time information on visual phenomena but independent of their identity could be regarded as part of the evidence that time is void of phenomena and is therefore given a priori (i.e., time is the form of the internal sense; Kant, 1998).

## Supplementary Material

Supplementary Material

## Data Availability

The data obtained through the behavioral and decoding analyses, as well as the analysis code itself, are available at https://osf.io/e9g5f/.

## Footnotes

* Even after performing multiple comparisons using the Benjamini-Hochberg correction including the hippocampus, left MFG, and LOC, the right OFC was shown to be statistically significant (*p* = 0.039).
