# Supplementary material for: Neural representations of visual statistical learning based on temporal duration
Source: Imaging Neurosci (Camb). 2025 Sep 3;3:IMAG.a.135. doi: 10.1162/IMAG.a.135 (PMC12409740; doi:10.1162/IMAG.a.135)
Supplement: Supplementary Material [file IMAG.a.135_supp.pdf]

Supplementary Materials Online for

Neural Representations of Visual Statistical Learning Based on Temporal Duration

Sachio Otsuka, Jun Saiki

Graduate School of Human and Environmental Studies, Kyoto University

* Sachio Otsuka, Graduate School of Human and Environmental Studies, Kyoto University, Yoshida-nihonmatsu-cho, Sakyo-ku, Kyoto 606-8501, Japan.

Email: otsuka.sachio.8a@kyoto-u.ac.jp

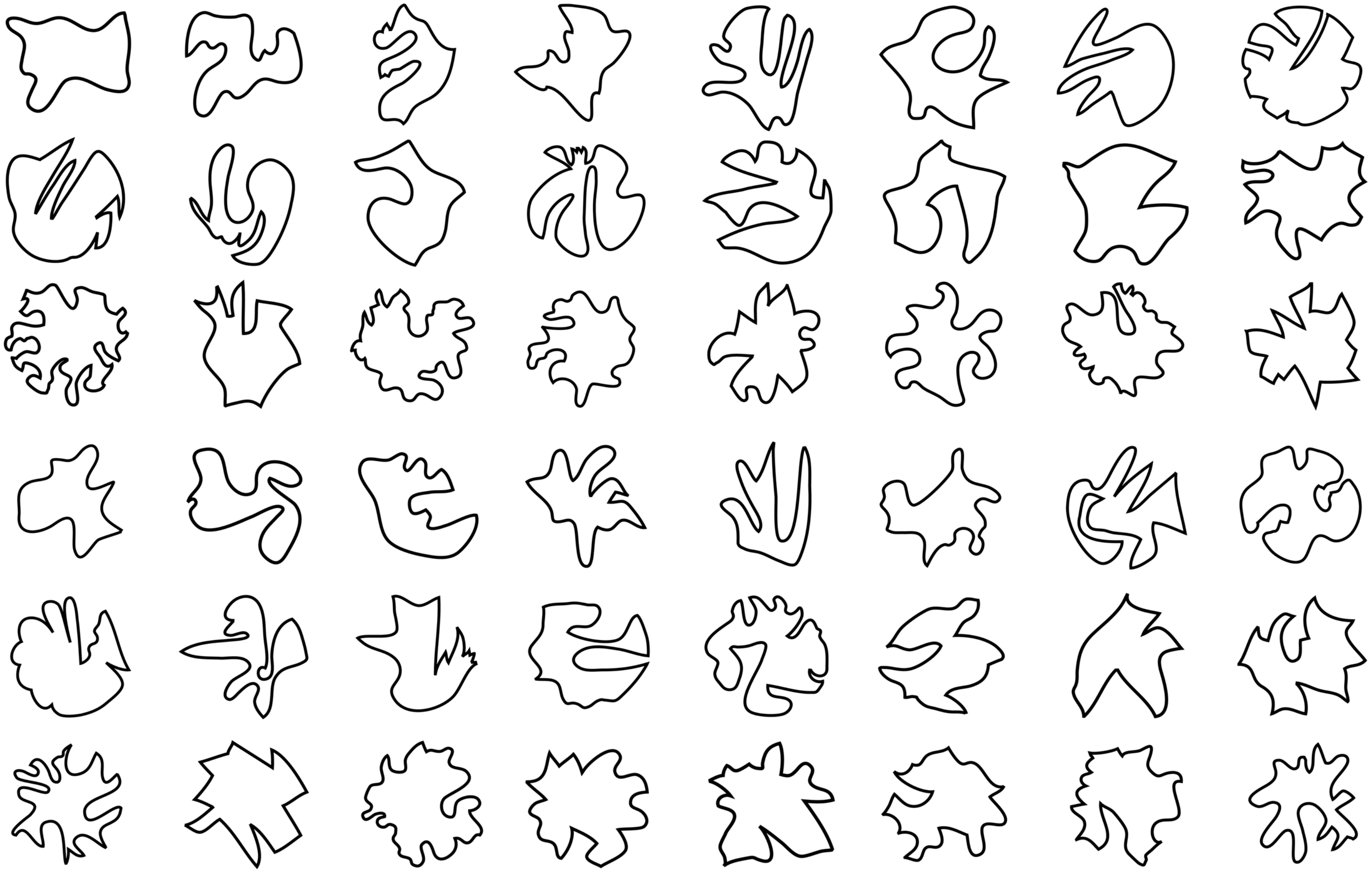

Supplementary Figure 1. Visual shapes used in this study selected from the stimulus set described by Endo et al. (2003).

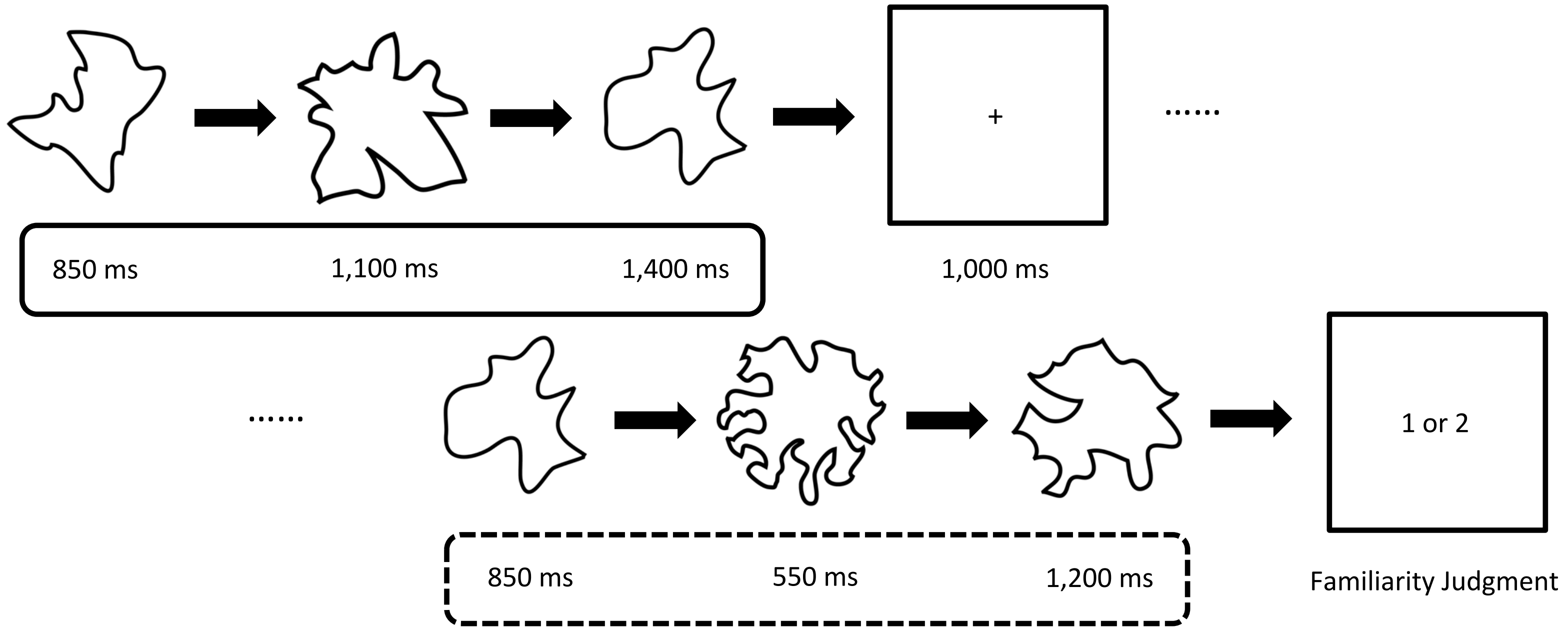

Supplementary Figure 2. Examples from a two-alternative forced-choice familiarity-decision task conducted outside the MRI scanner. These stimuli were selected from the stimulus set described by Endo et al. (2003). A solid line square represents a structured timing triplet based on the prior familiarization scan. A dashed line square represents a foil constructed from durations of three different structured triplets.

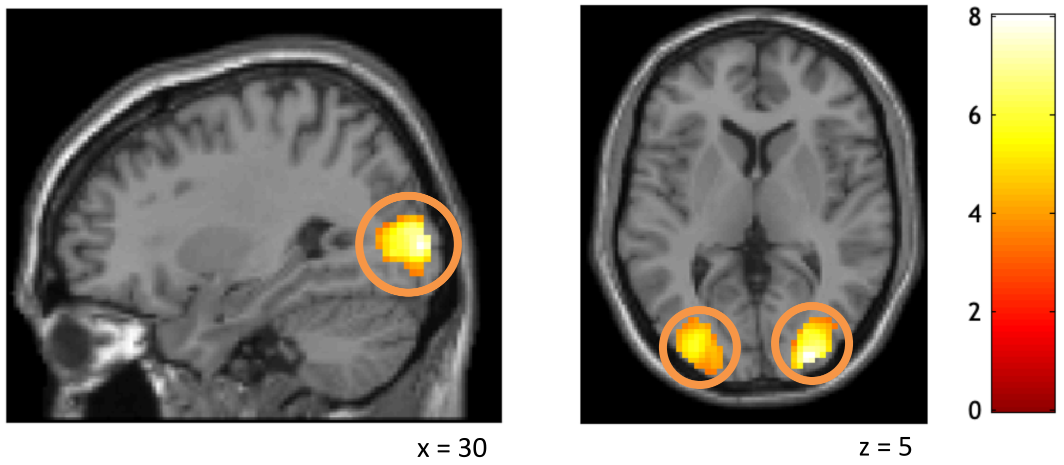

Supplementary Figure 3. Results of a localizer scan. The lateral occipital cortex exhibits stronger responses to line drawings than to faces (MNI coordinates, right: 30, –91, 5; $p < .001$ corrected; $k = 290$; peak $t = 8.00$; left: –21, –91, 4; $p < .001$ corrected; $k = 887$; peak $t = 6.31$).

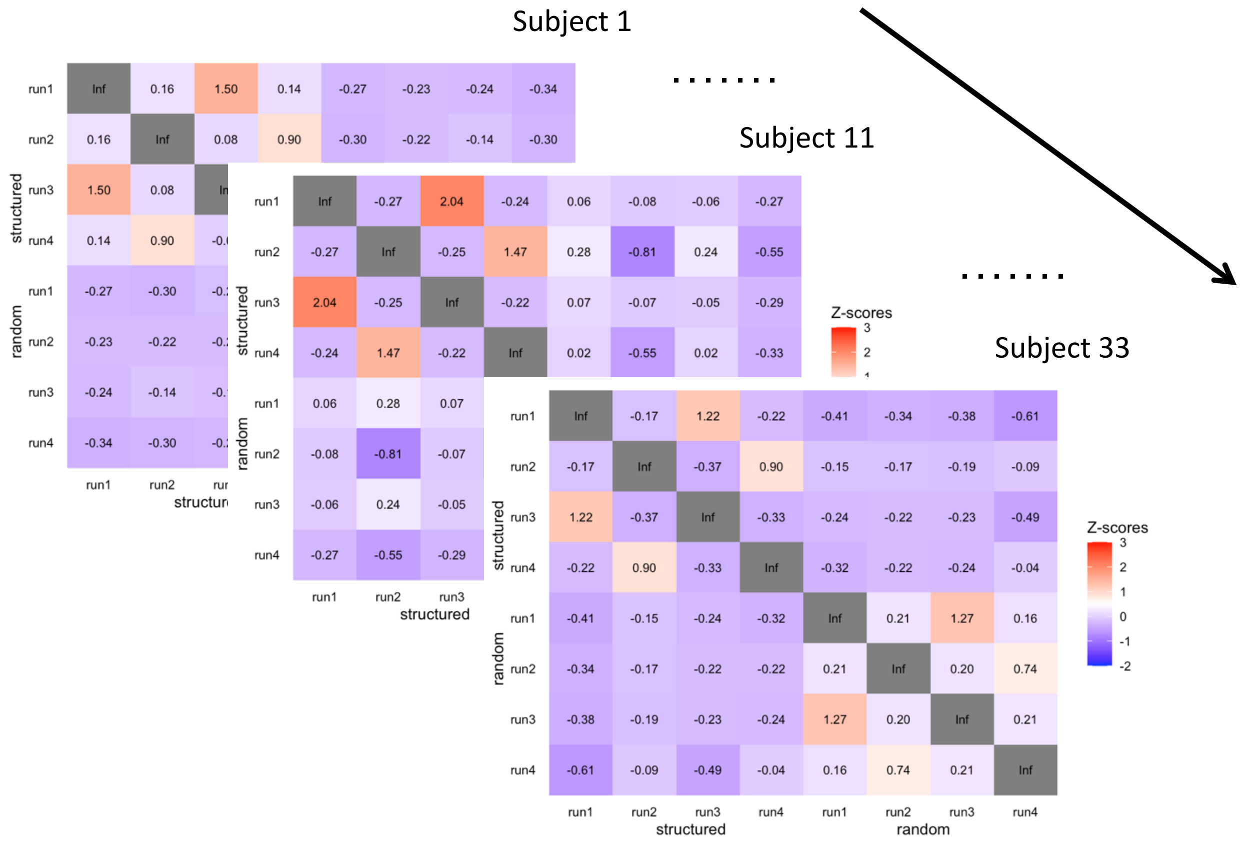

Supplementary Figure 4. Z-scored correlation matrices of fMRI data in the right OFC during the familiarization scan.

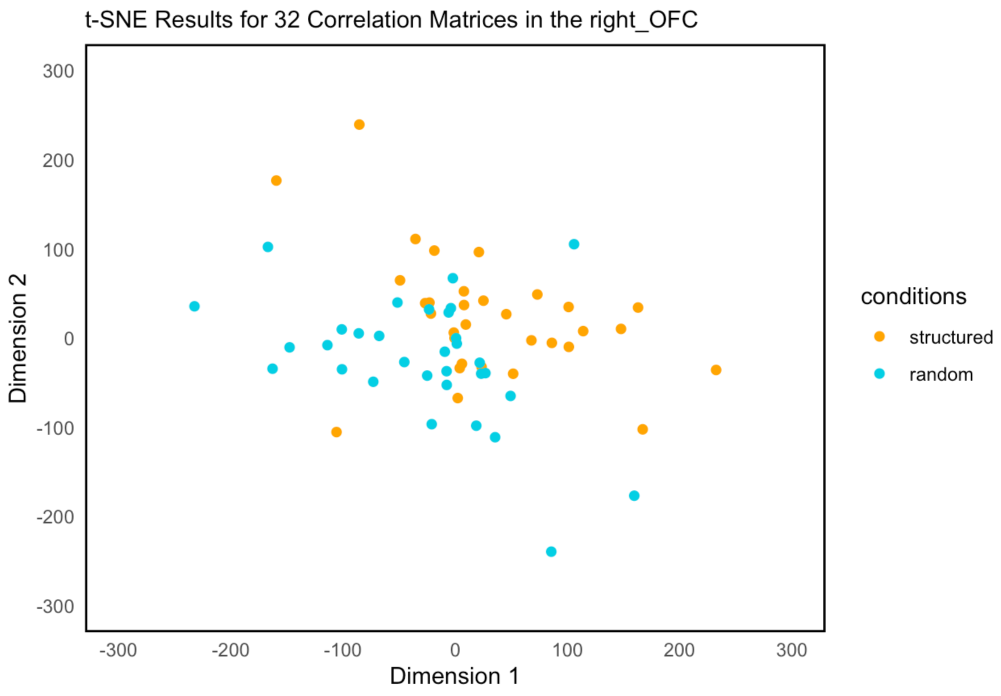

Supplementary Figure 5. Visualization of fMRI data as a function of the structured and pseudo-random conditions in the right OFC during the familiarization scan using t-SNE. Each point represents an individual score.

Supplementary Table 1. Durations of structured timing triplets for each participant (unit: seconds).

sub_ID	structured_1-1	structured_1-2	structured_1-3	structured_2-1	structured_2-2	structured_2-3	structured_3-1	structured_3-2	structured_3-3	structured_4-1	structured_4-2	structured_4-3
1	1.35	1.15	0.6	0.5	0.9	1.45	0.7	1.55	1	1.65	1.25	0.8
2	1.55	0.9	0.8	1.15	1.45	0.5	0.6	1	1.35	1.25	0.7	1.65
3	1.5	1.2	0.65	0.95	1.4	0.85	1.05	1.6	0.75	1.1	0.55	1.3
4	0.85	1.1	1.4	1.05	0.55	1.3	0.75	1.6	1.2	0.65	0.95	1.5
5	0.7	1.15	1.35	1.45	1.25	0.8	0.5	1.65	1	0.6	1.55	0.9
6	1.25	1.65	0.7	1.35	1.15	0.6	1.55	0.8	0.9	0.5	1	1.45
7	1.1	0.55	1.6	1.4	0.85	1.2	0.95	0.75	1.3	1.05	0.65	1.5
8	0.95	1.6	0.65	1.2	1.5	0.85	1.1	1.3	0.55	1.4	0.75	1.05
9	0.8	1.55	1.15	1.35	1.25	0.6	1.45	0.9	0.7	1	1.65	0.5
10	0.9	0.7	1.65	0.6	1.15	1.35	1	1.45	0.5	1.55	0.8	1.25
11	1.3	1.2	0.65	0.95	0.75	1.6	0.55	1.5	1.05	0.85	1.4	1.1
12	1.6	0.95	0.55	0.85	1.05	1.3	1.4	1.2	0.65	1.5	0.75	1.1
13	0.6	1.25	1.35	0.9	1.65	0.7	1.15	1.45	0.8	0.5	1	1.55
14	1.25	0.7	1.55	1.45	1	0.5	0.8	1.15	1.35	0.6	1.65	0.9
15	0.85	1.5	1.1	1.3	0.55	1.05	0.95	1.4	0.75	0.65	1.2	1.6
16	1.2	1.4	0.85	0.95	1.5	0.55	0.75	1.3	1.05	1.1	0.65	1.6
17	1.55	0.8	0.9	0.7	1.35	1.15	0.6	1.25	1.45	0.5	1.65	1
18	0.9	0.7	1.65	1.25	1.55	0.6	0.5	1	1.45	1.15	1.35	0.8
19	1.6	0.85	1.05	1.3	0.95	0.55	1.4	0.65	1.2	1.5	1.1	0.75
20	0.75	1.2	1.6	0.55	1.5	1.1	1.4	0.85	0.95	1.05	0.65	1.3
21	1.65	1.25	0.7	1.35	0.9	0.8	0.5	1.45	1	1.15	1.55	0.6
22	1.45	1.15	0.5	0.9	1.65	0.6	0.7	1.25	1.35	1	1.55	0.8
23	1.05	0.65	1.3	1.2	1.5	0.75	0.55	1.1	1.6	0.95	0.85	1.4
24	0.95	1.6	0.75	0.65	1.2	1.4	0.85	1.5	1.1	1.05	1.3	0.55
25	1.45	1.25	0.6	0.9	0.8	1.65	1.35	1.15	0.7	0.5	1	1.55
26	0.5	1.35	1.25	0.8	1	1.55	0.7	0.9	1.65	1.45	1.15	0.6
27	0.95	0.85	1.3	1.05	0.55	1.5	1.6	0.65	1.2	1.4	0.75	1.1
28	1.6	0.75	0.95	0.85	1.05	1.3	1.5	0.55	1.2	0.65	1.1	1.4
29	1.25	1.35	0.6	0.5	1.45	1	0.8	1.55	1.15	0.7	0.9	1.65
30	0.8	0.9	1.35	0.6	1.55	1	1.65	1.25	0.7	1.15	1.45	0.5
31	1.4	1.2	0.85	0.75	1.1	1.5	1.05	1.6	0.55	0.65	1.3	0.95
32	0.95	0.65	1.6	0.55	1.1	1.5	1.3	1.05	0.85	1.2	0.75	1.4
33	0.8	1.45	1	1.25	0.5	1.35	0.6	1.65	1.15	0.7	0.9	1.55
34	1.35	0.7	0.9	1.45	0.5	1.15	1.55	1.25	0.6	0.8	1	1.65
35	1.5	1.1	0.55	0.75	1.4	1.05	0.65	0.95	1.6	0.85	1.2	1.3
36	1.2	1.5	0.85	0.55	1.1	1.6	1.3	0.65	1.05	0.75	1.4	0.95

Supplementary Table 2. Durations of pseudo-random blocks for each participant (unit: seconds).

sub_ID	Durations of pseudo-random blocks in the first set				Durations of pseudo-random blocks in the second set				Durations of pseudo-random blocks in the third set			
1	0.55	0.85	1.05	1.5	0.65	0.75	1.1	1.6	0.95	1.2	1.3	1.4
2	0.75	1.05	1.1	1.5	0.65	0.85	0.95	1.3	0.55	1.2	1.4	1.6
3	0.6	0.8	1.45	1.65	0.7	0.9	1	1.35	0.5	1.15	1.25	1.55
4	0.8	1	1.25	1.55	0.5	0.6	1.45	1.65	0.7	0.9	1.15	1.35
5	0.55	0.65	1.4	1.6	0.85	0.95	1.2	1.5	0.75	1.05	1.1	1.3
6	0.55	0.75	0.85	1.3	0.95	1.05	1.4	1.6	0.65	1.1	1.2	1.5
7	0.8	0.9	1	1.25	0.6	0.7	1.35	1.45	0.5	1.15	1.55	1.65
8	0.5	1.25	0.7	1.65	0.9	1.15	0.8	1.45	1.55	1.35	0.6	1
9	0.75	1.1	1.3	1.5	0.65	0.85	1.2	1.6	0.55	0.95	1.05	1.4
10	0.65	0.85	1.3	1.4	0.55	0.95	1.2	1.5	0.75	1.05	1.1	1.6
11	0.6	0.8	1.15	1.45	0.5	1	1.55	1.65	0.7	0.9	1.25	1.35
12	0.5	0.8	1	1.15	0.6	0.7	0.9	1.35	1.25	1.45	1.55	1.65
13	0.75	0.85	1.05	1.2	0.65	1.1	1.4	1.6	0.55	0.95	1.3	1.5
14	0.95	1.05	1.2	1.6	0.75	1.1	1.4	1.5	0.55	0.65	0.85	1.3
15	0.5	1.35	1.45	1.65	0.7	0.9	1.25	1.55	0.6	0.8	1	1.15
16	0.8	0.9	1	1.65	0.6	1.15	1.25	1.45	0.5	0.7	1.35	1.55
17	0.65	1.05	1.1	1.5	0.55	0.95	1.2	1.3	0.75	0.85	1.4	1.6
18	0.55	1.05	1.3	1.4	0.65	0.95	1.1	1.5	0.75	0.85	1.2	1.6
19	0.6	0.8	1.35	1.45	0.5	1.15	1.55	1.65	0.7	0.9	1	1.25
20	0.6	1	1.45	1.65	0.5	0.7	0.9	1.35	0.8	1.15	1.25	1.55
21	1.05	1.3	1.4	1.6	0.85	0.95	1.1	1.5	0.55	0.65	0.75	1.2
22	0.55	0.95	1.05	1.1	0.85	1.2	1.3	1.4	0.65	0.75	1.5	1.6
23	0.5	0.9	1	1.35	0.6	0.7	0.8	1.65	1.15	1.25	1.45	1.55
24	0.6	0.9	1	1.15	0.5	0.8	1.25	1.55	0.7	1.35	1.45	1.65
25	0.55	0.85	0.95	1.05	0.75	1.4	1.5	1.6	0.65	1.1	1.2	1.3
26	0.85	1.1	1.2	1.4	0.65	0.75	1.05	1.3	0.55	0.95	1.5	1.6
27	0.6	1	1.55	1.65	0.5	1.25	1.35	1.45	0.7	0.8	0.9	1.15
28	0.7	1.15	1.35	1.55	0.6	0.8	1	1.65	0.5	0.9	1.25	1.45
29	0.75	0.85	1.05	1.1	0.55	1.3	1.5	1.6	0.65	0.95	1.2	1.4
30	0.85	1.4	1.5	1.6	0.55	0.65	0.75	1.3	0.95	1.05	1.1	1.2
31	0.5	0.6	0.7	1.35	1	1.25	1.45	1.55	0.8	0.9	1.15	1.65
32	0.7	0.8	1.35	1.55	0.5	0.6	1.45	1.65	0.9	1	1.15	1.25
33	0.75	0.85	1.05	1.1	0.65	0.95	1.3	1.5	0.55	1.2	1.4	1.6
34	0.55	0.75	1.2	1.3	0.65	0.85	0.95	1.6	1.05	1.1	1.4	1.5
35	0.7	1	1.45	1.55	0.8	1.15	1.25	1.35	0.5	0.6	0.9	1.65
36	0.6	1	1.45	1.55	0.5	1.15	1.25	1.35	0.7	0.8	0.9	1.65
